# Commencement of the Baltimore College of Dental Surgery

**Published:** 1858-04

**Authors:** 


*Commencement of the Baltimore College of Dental Surgery.*—The following gentlemen were admitted to the degree of Doctor of Dental Surgery, at the last commencement of the Baltimore College of

Dental Surgery. The residence and subject of Thesis of each graduate is annexed to his name:

Fernando Zayas Bazan, Cuba, Salivary Calculus.

Samuel Henry Beard, South Carolina, Origin and Development of the Teeth.

Armand Francis Bignon, M. D., Georgia, New Method of Mounting Continuous Gum Work.

Juan Nepomuceno Boza, Cuba, Preservation of the Natural Teeth.

Carver Willis Brown, Virginia, Inflammation.

Charles Wm. Cadden, M. D., Maryland, Odontalgia

Alonzo Lucius Carter, M. D., Switzerland, Pseudoplasmata.

Henry Clarke, Maryland, Dental Caries.

Luis Magin Diaz, Cuba, Anatomy of the Mouth.

Joseph Smith Dodge, Jr., M D., New York, Dental Histology.

Nath. Heyward Gibbes, M. D., South Carolina, Caries.

Edward Daniel Hamner, Virginia, Progress of Dental Science.

Middleton Stuart Hanckel, M. D., South Carolina, Caries of the Teeth.

Thomas Oliver Hills, District of Columbia, Adhesive Gold Foil.

Cornelius Searle Hurlbut, Massachusetts, Filling Teeth with Adhesive Foil.

Thornton William Tomlinson, Virginia, Diseases of the Maxillary Sinus.

Vines Edmund Turner, North Carolina, Odontalgia.

James Ghoulson Russell, M. D., Missouri, Hereditary Transmission of Disease.

George Porter Woodbury, Massachusetts, Importance of Preserving the Teeth.

